# Minimal sternotomy and transaortic access for transcatheter aortic valve implantation: 30-day outcomes in a single centre

**DOI:** 10.1186/1749-8090-8-S1-O318

**Published:** 2013-09-11

**Authors:** G Coletti, G De Cicco, G Di Matteo, C Fiorina, C Fucci, D Maffeo, F Ettori

**Affiliations:** 1 Cardiac Surgery Unit, Civic Hospital, Brescia, Italy; 2 Cardiac Catheterization Laboratory, Cardiothoracic Department, Civic Hospital, Brescia, Italy

## Background

Transcatheter Aortic Valve Implantation (TAVI) represents a new reasonable therapeutic approach in patients with severe aortic stenosis and a high risk for conventional surgery. The most common approach for TAVI remains the peripheral arteries, but in the recent years a transapical and transaortic approaches (TaoTAVI) have been described. In this context, we report our experience with TAVI adopting a direct ascending aorta access via minimal sternotomy (MS).

## Methods

Between October 2010 and May 2013, 23 patients (16 females) underwent TaoTAVI. In 21 of these patients the procedure was necessary because of a severe stenotic disease of peripheral arteries; in the remaining 2 patients, because of the presence of severe plaques projecting into the aortic lumen. Mean age was 83.3±6.8, mean log EuroSCORE II and STS score were 10.1±5.5 and 12.5±12.5, respectively. Procedure was performed by a multidisciplinary team. After a minimal midline sternotomy extended to the second right intercostal space, a CoreValve aortic prosthesis (CoreValve, Medtronic, Minneapolis, USA) was retrogradely implanted.

## Results

No death occurred in the operating theatre, whereas one patient died on the second post-operative day because of cardiac arrest. No significant aortic paravalvular leak post implantation, aortic dissection, annulus rupture or cardiac tamponade were observed. Most importantly, no stroke occurred. All the patients were asymptomatic and turned to relatively normal life and no further increasing of leak degree was detected at one month after discharge.

## Conclusion

Our initial experience suggests TaoTAVI via MS is a safe and feasible technique offering a new option for the patient in which the TAVI approach via peripheral arteries is unusable. Although further studies are needed for a greater confirmation, MS with direct aortic access for TAVI could become a reasonable option for patients unsuitable for a peripheral arteries approach.

